# Deep Profiling of the Aging Proteome Depicts Neuroinflammation, Synaptic Function, and Phosphorylation in an Accelerated Alzheimer's Disease Cell Model

**DOI:** 10.1016/j.mcpro.2025.101490

**Published:** 2025-12-17

**Authors:** Emma Gentry, Md Tarikul Islam, Huijing Xue, Kan Cao, Peter Nemes

**Affiliations:** 1 Department of Cell Biology & Molecular Genetics, University of Maryland, College Park, Maryland, USA; 2 Department of Chemistry & Biochemistry, University of Maryland, College Park, Maryland, USA

**Keywords:** aging, Alzheimer’s disease, neuroinflammation, mass spectrometry, proteomics, profiling, biomarkers

## Abstract

Alzheimer's disease (AD) is an age-associated neurodegenerative disorder characterized by amyloid plaques, tau hyperphosphorylation, and synaptic dysfunction. Most available cellular AD models lack aging features, limiting their ability to recapitulate key pathological mechanisms. Here we applied high-resolution mass spectrometry-based multiplexed proteomics and phosphoproteomics in a discovery setting to characterize an accelerated AD (acAD) model that combines amyloid precursor protein (APP) and presenilin (PSEN) mutations with progerin, an aging-associated Lamin A mutant that accelerates aging. Across four phenotypes (control, progerin, classic AD, and acAD), we identified 8279 proteins, quantified 6081 proteins, and detected phosphorylation dynamics. Relative to the classic model, acAD exhibited broader proteome remodeling, including amplified downregulation of synaptic and cytoskeletal proteins, upregulation of transcription and translation machinery, and pathway-level changes in neuronal signaling, mitochondrial dynamics, and neuroinflammation. Phosphoproteome analysis revealed widespread changes in RNA-binding and cytoskeletal proteins, aligning with recent data from two murine AD models. These findings show that acAD captures canonical AD phenotypes while uniquely modeling age-related inflammation and phosphorylation, providing a resource to accelerate studies of proteome-level mechanisms of AD progression and to inform strategies targeting cytoskeletal and inflammatory pathways.

## Highlights

• High-resolution proteomics quantified >6000 proteins among four aging cell models.
• The acAD model amplified synaptic loss, translation machinery, and inflammation.
• Phosphorylation patterns in acAD aligned with murine AD systems.

## Graphical Abstract

**Figure:**
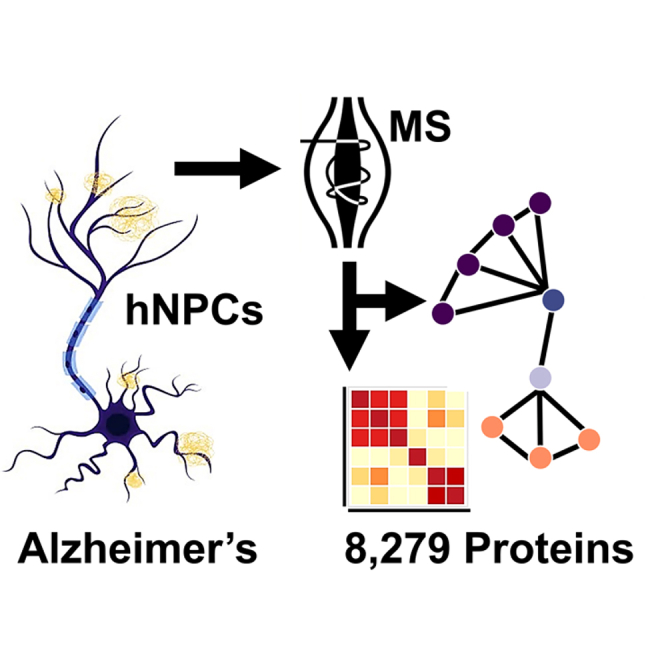

## In-Brief Statement

We applied HRMS-based multiplexed proteomics to characterize an accelerated Alzheimer’s disease cell model, uncovering amplified synaptic and cytoskeletal remodeling and phosphoproteomic shifts that mirror murine AD models.

At present, Alzheimer's disease (AD) involves coordinated disruption of memory, basic life skills, and independence, eventually ending in death. In 2024, 6.9 million Americans were affected, with these numbers rising as life expectancy increases (1). Treatment options for AD are currently insufficient. Several drugs, such as cholinesterase inhibitors and N-methyl-D-aspartate antagonists, seek to alleviate symptoms but do not alter the course of the disease (2, 3). New anti-amyloid antibody treatments have shown mixed success at slowing the disease, not reversing it, and only work if used early in the disease progression (4, 5). AD is a genetically complex disease, falling into two categories: familial AD with dominant mutations in APP and PSEN1 genes, and sporadic AD with risk factors such as APOE4 (6). A common major risk factor for AD is age.

AD pathology is classically identified by two protein aggregates: extracellular amyloid plaques and intracellular tau neurofibrillary tangles (7, 8, 9, 10). Beta amyloid is formed by the cleavage of amyloid precursor protein (APP) (11). Most familial AD is caused by mutations in either the APP gene or the complex cleaving it, the presenilins (PSEN1/2) (6). Compared to amyloid plaques, the levels of tau neurofibrillary tangles correlate better with AD symptoms and disease progression (12). Tau interacts with and stabilizes microtubules in the axons of neurons. It is heavily post-translationally modified by phosphorylation and methylation even in healthy controls (10). In AD, its heavy phosphorylation leads to aggregate formation, preventing its normal binding to the microtubules (10).

A multitude of AD models have been generated in animals and cell cultures. Both types of models usually struggle to recapitulate both amyloid plaque and tau pathology (13). Murine APP does not form aggregates because of three key sequence differences, even though homology is otherwise high (13). Murine models typically generate a phenotype with a high load of APP or multiple dominant familial AD mutations (13). Two common models are NLGF (14) and 5xFAD (15), containing three and five familial AD mutations in the APP gene, respectively. The disadvantages of AD mouse models have renewed interest in cell culture models of AD. Especially with the explosion of innovation in induced pluripotent stem cells (iPSC) technology, the capacity of cell culture to model human disease has grown tremendously (16). One challenge with fetal cell line and iPSC-based models is the lack of age markers (17). When iPSCs are reprogrammed, the epigenetic signature of age is erased, and the cells are phenotypically young (18). For AD, a disease of the aged, maintaining an aged phenotype in cell culture models is critically important to correctly model the disease progression.

We recently introduced an accelerated AD model (acAD) to address the missing age component of current cell culture models (19). We began with an established AD model using the Swedish and London amyloid precursor protein (APP) mutations and the ΔE9 presenilin 1 (PSEN1) mutation (20), referred to here as the *classic model* (CL). Then, a mutant form of Lamin A was incorporated, a component of the nuclear lamina. This protein, called progerin, is the cause of Hutchinson-Gilford Progeria Syndrome, or HGPS (21). HGPS systemically models premature aging, with thin skin, hair loss, and cardiovascular disease usually leading to death by the teen years (22). In natural aging, progerin accumulates in cells as well, in lower amounts (23, 24, 25, 26, 27). Progerin has been used in other disease models to capture the aging factor, such as in Parkinson's disease (17). Using progerin, the acAD model displayed robust plaque formation and tau phosphorylation with a dramatically reduced timeline from months to 4 weeks (19). Additionally, markers of neuronal and astrocytic distress, such as cell cycle reentry, are upregulated compared to CL, suggesting a more advanced disease state. Fully elucidating the molecular mechanisms behind the accelerated AD phenotype will shed light on how the incorporation of age affects AD progression.

The proteome is an efficient measure of the molecular phenotype. Studies surveying classical protein markers reported significant changes in neuroinflammation, mitochondrial function, and synaptic integrity during AD progression (28, 29). A computational proteomics network analysis has revealed neuroinflammatory pathways and protein candidates associated with AD during different stages of disease progression (30). To understand disease progression, global transcriptomic profiling from a large cohort of patients with AD has revealed extensive changes in gene expression related to synaptic transmission, metabolism, and cell cycle during the disease progression (31). With dynamic expression and posttranslational modifications (PTMs), such as phosphorylation, altering function, the proteome captures a snapshot of pathways to appreciate molecular cellular states.

High-resolution mass spectrometry (HRMS) allows for quantifying thousands of different proteins and their PTMs without needing functional probes. These analyses usually pool millions of cells for enhanced molecular coverage. Screening of ∼4000 proteins using this technology has recently uncovered differential production for ∼1058 proteins in the cerebrospinal fluid (CSF) from AD patients (32). Differential proteome profiling among patient CSF samples revealed AD subtypes (32). In the context of AD, phosphorylation is an important PTM, with abnormal phosphorylation of proteins such as tau has implicated in disease progression. Phosphoproteomic profiling by HRMS can capture and quantify global molecular states but the approach typically requires enrichment from large cell populations due to rare concentration and limitations in detection sensitivity.

Here, we apply HRMS proteomics and phosphoproteomics to benchmark acAD against the classical AD model (CL), a nontransfected control (Nt), and the progerin-only reference (Pg). We further compare phosphoproteome changes with two murine AD models, NLGF and 5xFAD (33). To ensure rigor, we prospectively validated the analytical fidelity of our HRMS quantification in this system. We then interpret the proteome and phosphoproteome against canonical pathways and biomarkers to gauge functional impact. We focus on established AD signatures in neuroinflammatory, mitochondrial, and cell-cycle programs as orthogonal benchmarks for proteome reorganization. The resulting dataset provides a valuable resource for studies that aim to integrate cellular aging into disease modeling.

## Experimental Procedures

### Chemicals, Reagents, Solvents

LC-HRMS grade acetone, acetonitrile (ACN), and formic acid (FA) were from Thermo Fisher Scientific. All other chemicals were obtained as reagent-grade or higher from the same vendor, unless specified otherwise. Ammonium bicarbonate (AmBic) and ammonium hydroxide (NH_4_OH) were from Avantor. Dithiothreitol (DTT) and iodoacetamide (IAA) were brought from Sigma-Aldrich. Sodium dodecyl sulfate (SDS), sodium chloride (NaCl), and TRIS were obtained from Amresco (Solon, OH), and deoxycholate were from Alfa Aesar. Triton X-100, triethylammonium bicarbonate (TEAB), and TMT10-plex reagent (lot no. SG253269) were purchased from Thermo Fisher Scientific. The RIPA buffer was prepared to contain: 50 mM Tris pH 7.5, 150 mM NaCl, 0.1% SDS, 1% Triton X-100, and 0.5% deoxycholate (source above). The ethylenediaminetetraacetic acid (EDTA)-free protease inhibitor tablets were from Roche (Basel, Switzerland).

### The hNPC Cell Culture

**Fig. 1:**
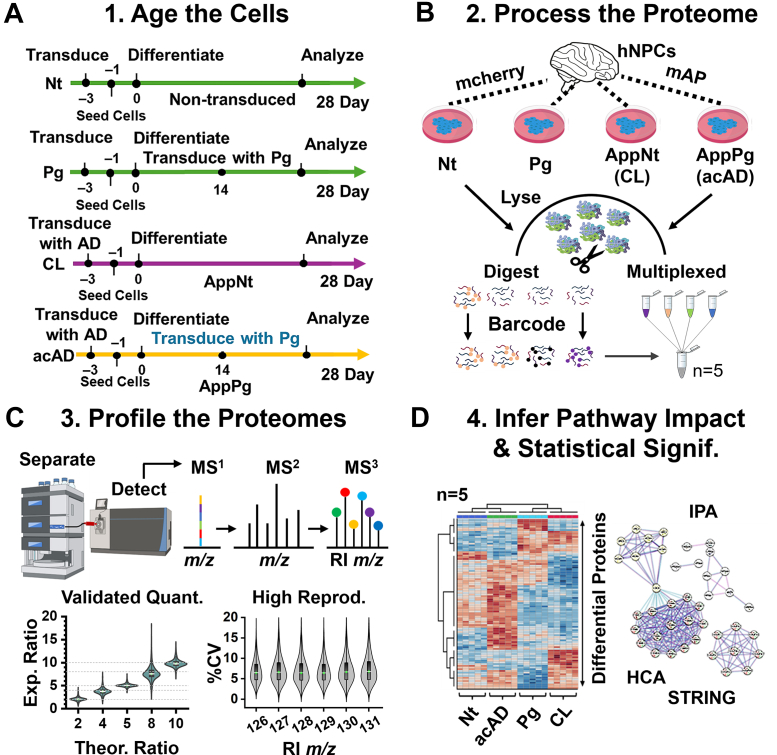
Experimental design and validation of proteome profiling in the accelerated Alzheimer's disease (acAD) model. *A*, schematic of cell model construction. Human neural progenitor cells (hNPCs) were transduced with either: (1) empty vector (nontransfected control, Nt), (2) progerin alone (Pg), (3) familial AD (fAD) mutations in APP and PSEN1 (classic model, CL), or (4) a combination of fAD mutations and progerin (acAD). fAD mutations were introduced 3 days prior to differentiation, and progerin was added on day 14. All lines were cultured for 28 days before analysis. *B*, workflow for multiplexed proteomics. Cell lysates were digested, labeled with tandem mass tags (TMT), fractionated by high-pH reversed-phase LC, and analyzed by nanoLC–HRMS (Orbitrap Fusion Lumos). Five biological replicates per phenotype were analyzed in technical duplicate. *C*, validation of quantification. Spike-in peptides labeled across six TMT channels confirmed accuracy and reproducibility of SPS-MS^3^ measurements, with <8% RSD. *D*, chemometric analysis. Unsupervised hierarchical cluster analysis (HCA) of the top differential proteins revealed distinct proteome signatures across Nt, Pg, CL, and acAD, supporting downstream pathway analyses (IPA, STRING).

The ReN VM immortalized human neural progenitor cells (hNPCs) from EMD Millipore were cultured in Geltrex-coated plates (Thermo Fisher Scientific A1413302). The proliferation media consisted of ReN maintenance media (EMD Millipore) supplemented with 20 ng/ml bFGF (R&D Systems) and 20 ng/ml EGF (Millipore Sigma). The cells were transduced with the mCherry vector or AD mutant plasmids as described below. To differentiate them, the cells were seeded at 8 × 10^5^ cells/well into 6-well plates coated with Geltrex, before further processing. The next day, the culture media was changed to the differentiation media: BrainPhys Neuronal Medium (STEMCELL) containing 10 U/ml heparin (Sigma-Aldrich) and B27 supplement (Thermo Fisher). The media was changed every 2 to 3 days until collection. The second transduction, *LMNA* or *LMNA*G608G, was performed on the 14th day as described below (Fig. 1*A*). The cells were collected using Accutase (StemCell), flash-frozen in liquid nitrogen, and stored at −80 °C until processing for HRMS.

### Lentivirus Packaging and Transduction

For lentivirus packaging, the two virus packaging vectors psPAX2 and pMD2.G (Addgene) were co-transfected along with the lentiviral plasmid into HEK293T cells with Fugene 6 (Promega). The media was changed at 12 h. The culture supernatants were collected at 48 h and 72 h post-transfection and then filtered (0.45 μm) to remove nonadherent cells. The purified supernatants were stored at −80 °C. To transduce ReN cells, the media was changed to include 8 μg/ml Polybrene (Santa Cruz Biotechnology), and the lentivirus was added. The media was changed 48 h later.

### Experimental Design and Statistical Rationale

We analyzed n = 5 biological replicates (BRs) per phenotype, each derived from ∼1 × 10^6^ cultured cells across the four phenotypes: Nt (negative control), Pg (negative control), CL (positive control), and acAD (experimental group). Each biological replicate was measured in technical duplicate, using a 5-plexing HRMS-barcoding strategy (see Proteome Processing). Raw ion counts were normalized to equal total peptide signal to control for minor loading differences. Batch effects across plexes were corrected before statistical testing. Normality was assessed with the Kolmogorov–Smirnov test. Two-sided Student's t-tests were applied, and *p* < 0.05 was considered significant.

### Proteome Processing

The cell pellets were thawed and lysed on ice for 20 min in the RIPA buffer supplemented with a protease inhibitor cocktail. The lysates were cleared by centrifugation at 20,000*g* for 15 min at 4 °C. Protein concentration in the supernatant was determined by bicinchoninic acid (BCA) assay. Proteins were reduced with dithiothreitol (DTT; 60 °C, 1 h), alkylated with iodoacetamide (IAA; room temperature, 20 min, dark), and the reaction was quenched with DTT. Proteins were precipitated with cold acetone (−20 °C, 12 h), collected by centrifugation (12,000*g*, 10 min, 4 °C), vacuum-dried, and resuspended in 50 mM AmBic. Tryptic digestion was performed at ∼1:50 protease-to-protein for 12 h at 37 °C. The peptides from the five biological replicates for each phenotype were labeled in five independent TMT plexes using six channels per plex (126, 127N, 128N, 129N, 130N, 131) from a TMT10-plex kit in 100 mM TEAB. Channel assignment within each plex was randomized. The labeled samples were pooled per plex, vacuum-dried, and reconstituted in 0.1% FA.

### Fractionation

To increase proteome depth, peptides were separated by high-pH reversed-phase LC into 72 fractions. TMT-tagged peptides were first trapped on a C18 guard column (InfinityLab Poroshell HPH-C18), then separated on an analytical C18 column (InfinityLab Poroshell 120 EC-C18) at room temperature using an Agilent 1260 Infinity II system. The LC operated at 0.5 ml/min with a 90-min gradient using mobile phase A (10 mM AmBic in water, pH ∼10) and mobile phase B (10 mM AmBic in 90% ACN, pH ∼10) as follows: 0% B, 0 to 17 min; 0 → 7% B, 17 to 18 min; 7 → 35% B, 18 to 75 min; 35 → 100% B, 75 to 76 min; 100% B, 76 to 80 min; 100 → 0% B, 80 to 86 min; hold at 0% B for 4 min to re-equilibrate the column. The fractions were concatenated into eight final pools to maximize orthogonality with nanoLC–HRMS analysis downstream. Each pool was vacuum-dried and reconstituted in 0.1% FA for analysis.

### NanoLC–HRMS Proteomics

Each peptide fraction was separated on a nanoLC system (UltiMate 3000, Thermo Fisher Scientific) and analyzed by electrospray ionization HRMS. The peptides were captured on a C18 trap column (Acclaim PepMap 100, 100 μm × 2 cm, 100 Å, 5 μm) in 100% A at 5.0 μl/min. Separation used a C18 μPAC analytical column (200 cm, Thermo Scientific; SN1100452) at 600 nl/min and 50 °C with a 240 min gradient of B as follows: 1%, 0 to 5 min; 1% → 7%, 5 to 20 min; 7% → 25%, 20 to 135 min; 25% → 32%, 135 to 160 min; 32% → 45%, 160 to 193 min; 45% → 75%, 193 to 200 min; hold 75%, 200 to 208 min; 75% → 2%, 208 to 210 min; equilibrate at 2% for 30 min. The nanoLC effluent was electrosprayed via a tapered-tip emitter (30/150 μm inner/outer diameter, Thermo ES542) at +2.3 kV.

The peptide ions were measured on an Orbitrap Fusion Lumos mass spectrometer (Thermo) operated in data-dependent acquisition (DDA). HRMS^1^ scans were acquired in the Orbitrap at 120,000 FWHM, max IT auto, AGC 5 × 10^3^, one microscan. Precursors were isolated with a 0.7 Th window and fragmented by collision-induced dissociation at 35% normalized collision energy (NCE) with helium as the collision gas. Tandem mass (MS^2^) spectra were detected in the ion trap (AGC target standard, max IT auto, one microscan). Quantification used SPS-HRMS^3^ targeting the top 10 MS^2^ fragments, with HCD in nitrogen at 55% NCE. Reporter ions (RIs) were detected in the Orbitrap at 50,000 FWHM (AGC target 300% normalized; max IT auto).

### Data Analysis

The HRMS data were processed in Proteome Discoverer 2.2 or 3.0 (Thermo). The HRMS spectra were searched with SEQUEST against the *Homo sapiens* proteome (20,380 genes, downloaded from UniProt database on 6/8/2021). The global proteome search parameters were: enzyme, trypsin; number of missed cleavages, two max; minimum number of high confidence unique peptides, 1; static modification, cysteine carbamidomethylation and lysine TMT10-plex; variable modification, methionine oxidation, N-terminus acetylation; maximum mass deviation allowed for precursor masses, 10 ppm; mass tolerance for tandem mass spectra, 0.6 Da. For phosphoproteomics, a separate search included phosphorylation (+79.966 Da) on serine, threonine, or tyrosine as an additional variable modification. All other parameters matched the global proteome search. Protein identifications are reported as protein groups (UniProt accession number), unless sufficient HRMS data were available to decipher proteoforms.

### Statistics and Pathway Analysis

#### Signal Processing

Peptide and protein identifications were filtered to <1% false discovery rate using a reversed-sequence decoy strategy. TMT quantification used the abundance of the RIs generated in MS^3^. Phosphosite localization confidence was assessed with the ptmRS node in Proteome Discoverer 3.0. Only sites with ptmRS probability >0.75 were retained for biological interpretation.

#### Quantification

To compare equal amounts of proteome digests, RI intensities were normalized to total peptide signal in Proteome Discoverer 2.2 (proteomics) or 3.0 (phosphoproteomics). Proteins were required to have signal/noise > 5 in at least three of the 5 BRs to enter downstream statistical and pathway analyses. To reduce inter-plex variation and improve consistency, RI intensities were log_10_ transformed and median normalized in Perseus 2.0.20.0. Data were then exported for batch-effect correction with ComBat across the five plexes, followed by downstream analysis in MetaboAnalyst 5.0 (34). Data normality was confirmed with the Kolmogorov–Smirnov test before applying two-sided Student's t-tests, with *p* < 0.05 considered significant.

#### Pathway Analysis

Gene Ontology term annotations were performed for *Homo sapiens* using PANTHER 19.0 (35). Pathway significance in Ingenuity Pathway Analysis (IPA 2024) was assessed by Fisher's exact test with *p* < 0.05. The IPA Z-score metric was used to predict pathway activation or inhibition following the vendor's recommendations.

### Safety

Chemicals and biological specimens were handled with the appropriate personal protective equipment, following standard safety protocols. Biological materials were disposed of in accordance with institutional regulations.

## Results

### Proteome Profiling

As illustrated in Figure 1*A*, we benchmarked the acAD model against CL (20, 36). In the acAD model, progerin (Pg), a mutant form of *LMNA* associated with premature aging, was additionally expressed beginning on day 14 of differentiation (19). To examine how Pg accelerates disease-relevant proteome changes, we profiled four phenotypes at day 28: Nt, Pg, CL, and acAD (Fig. 1*B*).

We designed a high-sensitivity quantitative strategy. The design (Fig. 1*B*) compared the four phenotypes in n = 5 BRs, each analyzed in technical duplicate. Although modern barcoding (e.g., TMTpro 32-plex) could accommodate all our 20 samples in a single experiment, we used a 4-plex layout to maximize sensitivity. Using fewer labeling channels increases ion counts per channel within the instrument's finite ion-storage capacity, thereby improving empirical sensitivity at the expense of throughput. This also allowed the use of the remaining 126 to 131 channels from a TMT10-plex kit. Data acquisition used SPS-MS^3^ quantification to minimize coisolation interference (37, 38).

Quantitative performance was benchmarked with a peptide spike-in mixed at defined ratios (1:2:4:5:8:10) and labeled across the 6 RI channels (*m/z* 126–131). The data were processed with log_10_ transformation, median centering, and ComBat batch correction (Experimental Procedures). This enabled direct comparison across replicates without a dedicated reference channel, preserving sensitivity per analyte channel. For interpretation, we required SI signal/noise >5, used *p* < 0.05 for significance, and applied a ±1.2-fold effect-size filter. In the validation run, the expected and observed RI ratios matched theoretical values with <8% relative standard deviation (RSD), confirming analytical fidelity (Fig. 1*C*).

Using this workflow, we quantified proteomes for Nt, Pg, CL, and acAD. We identified 8279 protein groups (Supplemental Table S1), of which 6081 were quantified in at least three of the 5 BRs above the signal-to-noise threshold (Supplemental Table S2). Hierarchical cluster analysis (HCA) assessed the global differences into four phenotypes. HCA of the top 100 most variable proteins resolved 4 clusters that corresponded to Nt, Pg, CL, and acAD after sample identities were revealed (Fig. 1*D*; close-up in Supplemental Fig. S1). These results indicate distinct proteomic signatures for each aging model.

### Synaptic, Cytoskeletal, and Translational Shifts

**Fig. 2:**
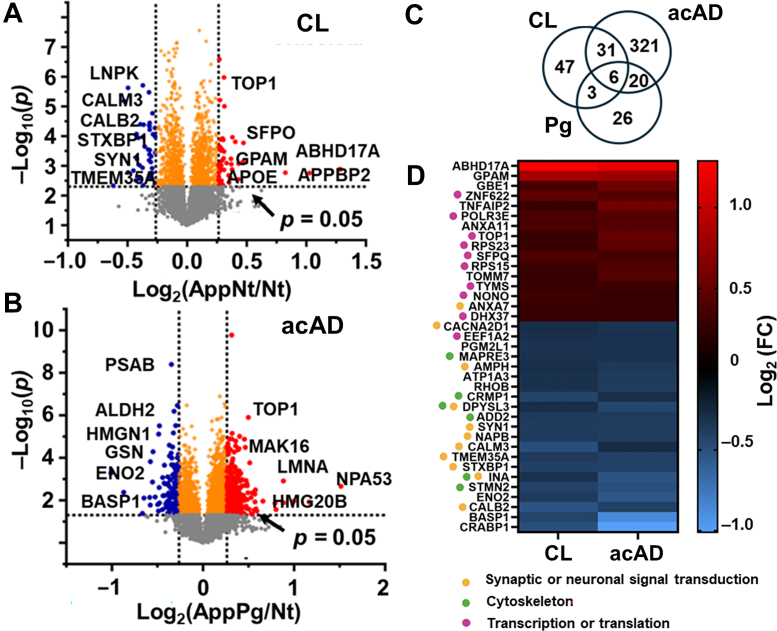
**Differential protein expression in the accelerated Alzheimer's disease (acAD) model.***A*, Volcano plot comparing the classic model (CL) to nontransduced control (Nt), showing 1199 significantly altered proteins (*p* < 0.05; red = upregulated, blue = downregulated). *B*, Volcano plot comparing acAD to Nt, identifying 1929 significantly altered proteins, indicating a broader proteomic shift. *C*, Venn diagram of proteins passing both statistical (*p* < 0.05) and fold-change (±1.2) thresholds. 37 proteins were common to both models, 341 were unique to acAD, and 55 were altered in the Pg condition. *D*, Heatmap of the 37 shared proteins, grouped by canonical function. Expression changes (log_2_ fold change vs Nt) were directionally concordant between CL and acAD, with many amplified in acAD. Functional categories include synaptic signaling (*orange*), cytoskeletal organization (*green*), and transcription/translation (*pink*).

The relative abundances and statistical significance of proteins in the classic and acAD models are summarized in Figure 2, *A* and *B*, with the Pg condition shown in Supplemental Figure S2. The summary of statistics is presented in Table 1, and quantitative details are available in Supplemental Table S3. Approximately 70% of proteins quantified by nanoLC–HRMS exhibited comparable abundance across phenotypes. In CL, 1199 proteins differed significantly (*p* < 0.05) relative to the Nt control, with 613 upregulated and 586 downregulated (Fig. 2*A*). The acAD model exhibited a broader shift, with 1929 differential proteins: 1365 were upregulated and 564 downregulated (Fig. 2*B*). The Pg showed 1070 proteins altered, with 443 increased and 627 decreased (Supplemental Fig. S2, Table 1). 465 proteins had significant enrichment changes: 87 in CL and 378 in acAD (Fig. 2*C*). Only 55 were differentially expressed in the Pg condition. These findings underscore the broader and more pronounced molecular changes in the acAD model.

**Table 1:** Differential proteins across the aging models

Sample	Differential	Upregulated	Downregulated	Differential	Upregulated	Downregulated
	Filtered to *p* < 0.05			Filtered *p* < 0.05 and FC > 1.2		
Pg	1070	443	627	55	30	25
CL	1199	613	586	87	46	41
acAD	1929	1365	564	378	258	120

Proteins showing significant changes relative to control are summarized for each phenotype (Pg, CL, acAD). Values are reported as total differential proteins (*p* < 0.05, two-tailed Student's *t* test) and as subsets passing both *p* < 0.05 and fold-change (FC) > ±1.2. Upregulated and downregulated counts are listed separately.

Across overlapping proteins altered in both classic and acAD models, expression patterns were directionally concordant, with many changes amplified in acAD (Fig. 2*D*). Notably, proteins involved in cytoskeletal regulation (e.g., MAPRE3, CRMP1, DPYSL3, ADD2, INA, and STMN2) and synaptic or signaling functions (e.g., CACNA2D1, AMPH, SYN1, NAPB, CALM3, TMEM35A, STXBP1, and CALB2) were consistently downregulated. Conversely, proteins associated with transcription and translation, including RNA-binding and ribosomal components (e.g., ZNF622, POLR3E, TOP1, RPS23, SFPQ, RPS15, TYMS, NONO, and DHX37), were significantly upregulated. These results indicate a coordinated reduction in neuronal structure and communication, accompanied by increased protein synthesis and gene expression activity, particularly in the acAD model.

### Impact on Protein-Protein Interaction Networks

**Fig. 3:**
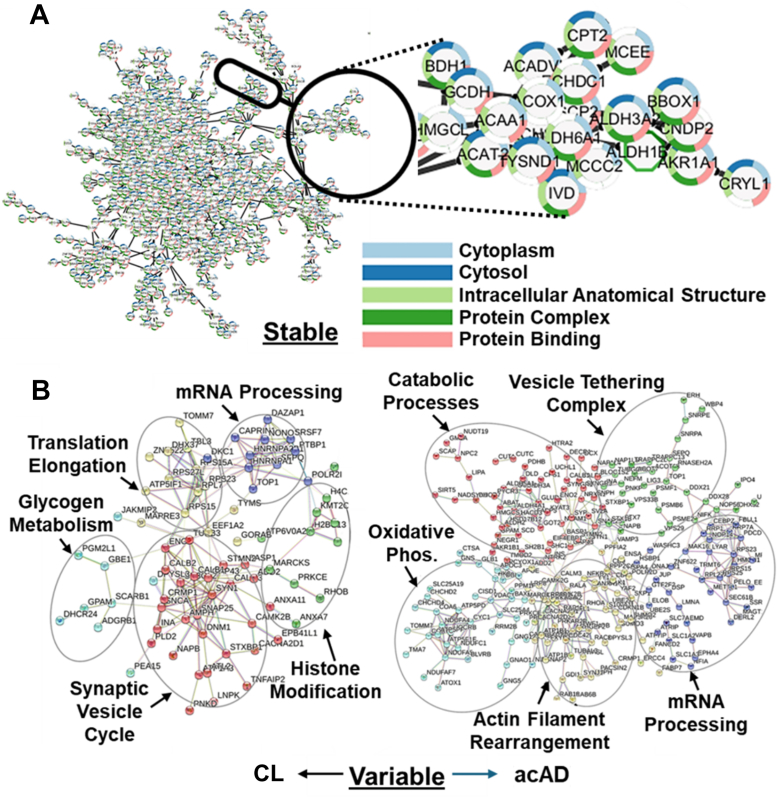
**Protein interaction networks altered in the accelerated Alzheimer's disease (acAD) model.***A*, STRING network of stably expressed proteins revealed dense, conserved clusters representing canonical housekeeping machinery, including translation and cytoplasmic organization. *B*, STRING network of differentially expressed proteins highlighted model-specific interaction patterns. CL showed changes in synaptic vesicle cycling and transcription/translation regulation, while acAD exhibited broader alterations involving oxidative phosphorylation, cytoskeletal remodeling, and RNA metabolism. Both AD models showed downregulation of synaptic proteins (e.g., synapsin-1, CaMKII, calmodulin), whereas the Pg condition primarily enriched nuclear processes such as DNA repair and lamin A processing. (See Supplemental Figs. S3 and S4 for close-up views.)

We used STRING to investigate protein–protein interactions and assess the functional protein networks affected in each model. The interactome constructed from stably expressed proteins revealed a dense, highly interconnected network composed of canonical cellular machinery involved in translation, cytoplasmic organization, and protein-binding complexes (Fig. 3*A*, close-up in Supplemental Fig. S3). These conserved components appeared to be largely stable across conditions, reflecting core housekeeping processes necessary for cell viability.

In contrast, the proteins with altered levels showed striking differences in predicted interaction patterns between the AD models (Fig. 3*B*, Supplemental Fig. S4). CL exhibited changes in clusters related to synaptic vesicle cycling and transcription/translation regulation. In the acAD, these differences were more pronounced, with additional alterations observed in clusters governing oxidative phosphorylation, cytoskeletal remodeling (e.g., actin filament dynamics), and RNA metabolism. These enhanced shifts suggest more extensive cellular reprogramming in the acAD condition, consistent with accelerated pathology. Both AD models exhibited downregulation of synaptic proteins, such as synapsin-1, CaMKII, and calmodulin, which implicates impaired vesicle trafficking and neurotransmission. In contrast, the Pg control showed no detectable changes in neuronal signaling modules and instead exhibited enriched interactions related to nuclear processes, such as DNA repair and lamin A processing (Supplemental Fig. S2*B*). Gene Ontology (GO) enrichment analysis (39) further supported these findings (Supplemental Fig. S5), highlighting significant disruptions in neuronal signaling and synaptic function in both AD models, particularly in acAD. These data suggest that the classic and acAD models share deficits in synaptic and metabolic pathways, with the acAD model exhibiting a more severe and multifaceted phenotype.

**Fig. 4:**
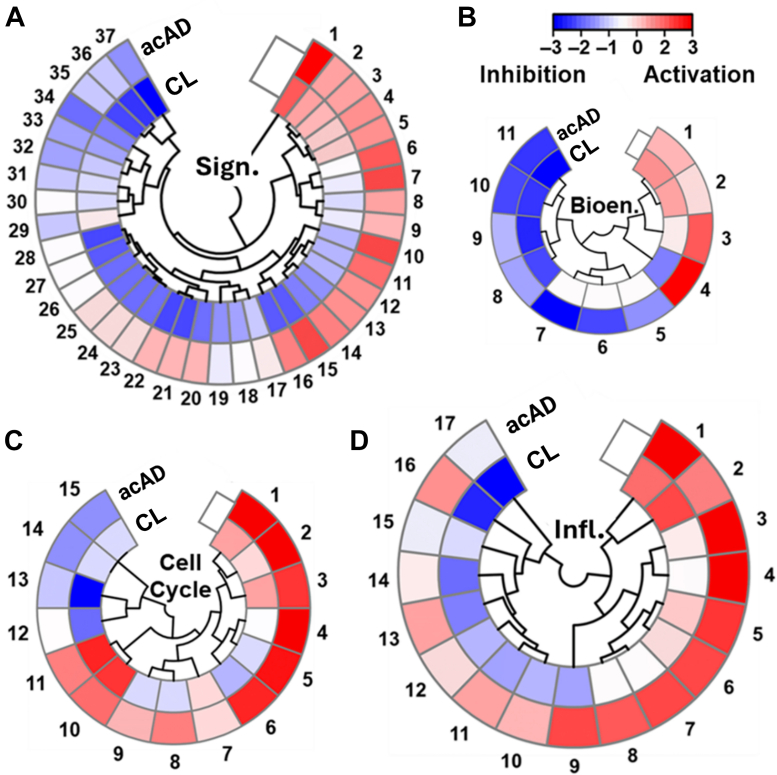
**Pathway enrichment analysis of proteomic changes in the Alzheimer's disease models.** Ingenuity Pathway Analysis (IPA) identified four major clusters among 6081 quantified proteins. *A*, Neuronal signaling and function, including glutamate/glutamine metabolism, AMPA receptor activation, and synaptogenesis signaling. *B*, bioenergetics, with activation of mitochondrial translation and mitophagy. *C*, cell cycle regulation, with global upregulation consistent with cellular stress responses. *D*, neuroinflammation, with widespread activation of interleukin signaling and senescence-associated pathways in acAD. Pathways were defined using a significance threshold of *p* < 0.05 (see Supplemental Table S4 for labels).

To contextualize proteomic changes within established biological processes, we compared acAD and CL through pathway enrichment analysis. Of the 6081 quantified proteins, Ingenuity Pathway Analysis (IPA) revealed four major clusters affected across models: neuronal signaling (Fig. 4*A*), bioenergetics (Fig. 4*B*), cell cycle regulation (Fig. 4*C*), and neuroinflammation (Fig. 4*D*). Bioenergetics and cell cycle showed similar results in both models, whereas there were differences in neuronal signaling and neuroinflammation between acAD and CL. In the neuronal signaling group (Fig. 4*A*), the acAD model promoted glutamate and glutamine metabolism (A5), glutamate binding to activation of AMPA receptors and synaptic plasticity (A23), synaptogenesis signaling (A24), ion channel transport (A31), and glutaminergic receptor signaling (A25). This suggests an activation of signaling, which concords with the hyperactivity seen in early-to mid-AD (40). A focused analysis of the bio-energetic pathways in Figure 4*B* reveals activation of mitophagy (B2) and mitochondrial translation (B4). This finding corroborates the elevated oxidative phosphorylation that our STRING analysis suggested with AD progression (Fig. 3*B*, close-up in Supplemental Fig. S4).

The cell cycle pathways (Fig. 4*C*) show global upregulation in the acAD model, another marker of cellular dysfunction (labeled C1–15 in Supplemental Table S4), since neurons only enter the cell cycle as a response to distress. The neuronal signaling pathways in Figure 4*D* reveal a widespread activation of proinflammatory interleukins (labeled D1–17 in Supplemental Table S4), including NF-κB and other senescence-associated secretory phenotype (SASP) factors in the acAD model. A version of this figure including the Pg control is available in Supplemental Fig. S2*C* (Supplemental Table S5). Notably, the progerin-only control did not explain the changes seen in acAD to neuroinflammation or neuronal signaling.

### Phosphorylation Dynamics

**Fig. 5:**
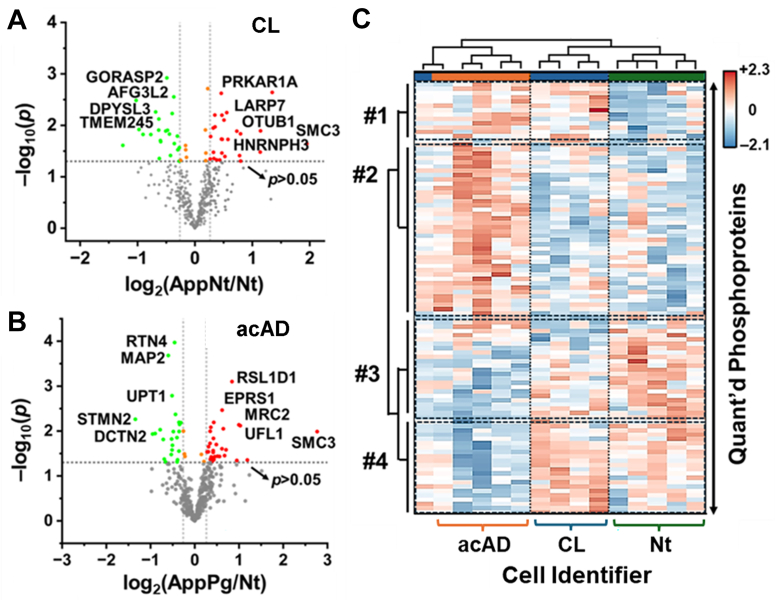
**Phosphoproteomic changes in the accelerated Alzheimer's disease (acAD) model.***A–B*, Volcano plots of phosphosites significantly altered (*p* < 0.05, fold change >1.2) in CL and acAD compared to Nt control, normalized to protein level. *C*, hierarchical clustering of the top 80 differentially abundant phosphosites revealed distinct molecular patterns among phenotypes. Replicates clustered tightly by condition, confirming reproducibility. Clusters highlighted phosphoproteins involved in cytoskeletal regulation, RNA metabolism, apoptotic pathways, and mitosis (see Supplemental Fig. S6 for extended analysis).

Although we did not enrich phosphopeptides by design, we reanalyzed the HRMS data to profile phosphorylation, a hallmark of AD. Hyperphosphorylation and subsequent aggregation of tau are hallmarks of AD (7). It is shown that the levels of tau neurofibrillary tangles align well with AD symptoms and disease progression (12). Tandem MS enabled site localization, and TMT reporter ions provided relative phosphopeptide abundance. Significant phosphosites vs. control are summarized in Figure 5, *A* and *B*. An unsupervised HCA heat map of the 80 most significant sites, one per protein, separated samples by phenotype (Fig. 5*C*; close-up in Supplemental Fig. S6). The proteins were clustered into four characteristic concentration profiles across the sample types (labeled **Clusters #1–4**; details in Supplemental Table S6).

These phosphoprotein profiles reveal dynamic molecular changes across AD development. Cluster #1 exhibited modest enrichment in CL and a stronger increase in acAD (Fig. 5*C*). Members included RNA processing factors such as U2AF2 and HNRNPH3 and the deubiquitinase USP10, consistent with altered RNA splicing and translational control during AD pathology. Proteins in Cluster #2 remained low in CL and Nt but increased in acAD. This group was enriched for cytoskeletal components, including MAPT (tau), LMNA, and SMC3. STRING analysis linked these proteins to aggrephagy, apoptotic execution phase, microautophagy, and apoptotic cleavage pathways. Activation of proteostasis and apoptotic programs is consistent with the clearance of tau and β-amyloid aggregates during progression. LMNA phosphorylated at Ser22 was elevated in acAD relative to control, consistent with lamin disassembly during mitosis (41). In agreement, IPA of the proteome supported cell-cycle reentry in acAD.

In contrast, Clusters #3 and #4 decreased in acAD relative to Nt alone (Cluster #3) or to both Nt and CL (Cluster #4). These sets included phospho-cytoskeletal proteins associated with growth cones, axons, and neuronal soma, which were reduced in acAD while remaining comparable between CL and Nt. The Pg condition did not differ from control in its phosphoproteome profile (Supplemental Fig. S2*D*), indicating that this changed phosphorylome phenotype is specific to acAD. Supplemental Figure S7 summarizes phospho-synaptic proteins quantified across CL and acAD and highlights shared interactors. Collectively, these patterns indicate impaired neuronal function in acAD, as expected in AD.

**Fig. 6:**
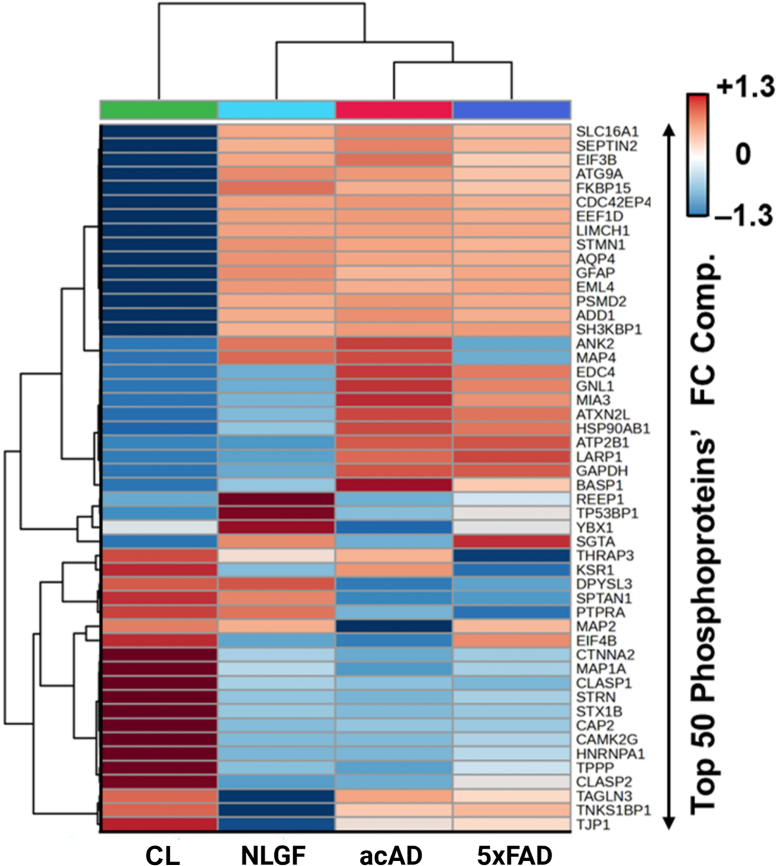
**Concordance of phosphorylation changes between acAD and murine Alzheimer's disease models.** Phosphorylation profiles from CL and acAD were compared with published datasets from 12-month-old NLGF and 5xFAD mice. Hierarchical clustering of the top 50 differentially phosphorylated proteins (*p* < 0.05, fold change >1.2, normalized to protein levels) grouped acAD with the two murine models, distinct from CL. The shared phosphosites included cytoskeletal and translation-associated proteins, underscoring the alignment of acAD with *in vivo* AD systems.

To compare the acAD phosphorylation changes with other AD models, we analyzed data from a recent study profiling phosphoproteomes in 12-month-old NLGF and 5xFAD mice relative to age-matched healthy controls (33). Figure 6 shows the top 50 proteins with differentially modified phosphorylation sites after normalization to protein level. HCA clustered the acAD model closely with the murine models, whereas CL did not. Among these top 50 differentially phosphorylated proteins, acAD matched at least one murine model at 43 sites. It aligned with both models at 28 sites and with a single model at 15 sites.

Among those 28 shared sites between acAD and two murine models, 15 sites exhibited increased phosphorylation relative to CL. These included cytoskeletal-associated proteins SEPTIN2, FKBP15, CDC42EP4, LIMCH1, STMN1, GFAP, EML4, and ADD1, as well as translation-related factors EIF3B and EEF1D. The remaining 13 sites showed reduced phosphorylation compared to CL. These included cytoskeleton-linked proteins TAGLN3, TJP1, CTNNA2, MAP1A, CLASP1, CLASP2, CAP2, and TPPP, along with regulators of phosphorylation such as TNKS1BP1 and STRN. Together, these changes point to convergent remodeling of cytoskeletal organization and translational control in acAD.

At 15 sites, acAD showed phosphosite changes that aligned with one of the two murine AD models. Increases were observed on proteins involved in protein processing, including MIA3 and HSP90AB1. Additional upregulated sites occurred on cytoskeletal-related proteins such as ANK2 and MAP4. Conversely, downregulated phosphosites were enriched in cytoskeletal components, including DPYSL3 and SPTAN1. These comparisons suggest that acAD recapitulates key complex phosphoproteomic shifts observed in *in vivo* models and further support the cytoskeletal remodeling identified by the STRING network analysis.

Extending beyond murine models, we finally compared the phosphorylation data to a human postmortem phosphoproteomic study (42). Of the 387 detected phosphoproteins in acAD, 283 overlapped with the postmortem dataset (73% match, Supplemental Fig. S8). This substantial overlap indicates concordance between our model and molecular changes observed in the patient brain during AD progression.

## Discussion

Our proteome-wide analyses demonstrate that the acAD model uniquely recapitulates age-associated neuroinflammation, characterized by broad upregulation of SASP and interleukin signaling (Fig. 2, Fig. 3, Fig. 4, Fig. 5, Fig. 6). These features extend beyond the classic AD model and arise despite the absence of phosphopeptide enrichment. Together with cross-model concordance in murine AD and overlap with human postmortem phosphoproteomes, these data position acAD to probe mechanisms and to benchmark interventions. We next consider mechanistic implications, study limitations, and translational use.

### Neuroinflammation

Conventional culture models often rely on highly proliferative, developmentally young cells, which can limit their ability to reproduce key features of classic AD pathology. The acAD model builds on a glia-inclusive classic coculture and imposes a systemic, multi-pathway aging driver by expressing Pg. To our knowledge, this is the first AD cellular model to incorporate such an aging factor (20). The resulting neuroinflammatory signature, together with the broader proteomic and phosphoproteomic changes reported here, outlines mechanisms that may underlie accelerated pathology in acAD.

Inflammation is a hallmark of aging and a critical component of AD neurodegeneration, with evidence for a causal contribution (43). Exogenous inflammatory cytokines exacerbate classic AD phenotypes in cell culture and mouse models (44, 45). Using validated, sensitive, quantitative HRMS, we find that the acAD culture exhibits a broad neuroinflammatory program, with marked upregulation of SASP and interleukin pathways compared to CL. The associated cellular stress is consistent with the more pronounced phenotype in acAD and with activation of cell cycle programs. Neurons are terminally differentiated and can reenter the cell cycle aberrantly under stress as part of attempted repair. Consistent with this, we observe widespread upregulation of cell cycle pathways (e.g., senescence and inflammation, Fig. 4), in agreement with our prior FACS-based assays (19).

### Cytoskeletal Remodeling

Quantitative proteomics and phosphoproteomics consistently implicate cytoskeletal remodeling as a central feature of acAD, highlighting tau destabilization, actin reorganization, and impaired axonal transport that converge on synaptic dysfunction (Fig. 2, Fig. 3, Fig. 5, Fig. 6). Tau stabilizes microtubules, and its dysfunction leads to cytoskeletal destabilization (10). Synaptic actin is disrupted in AD and contributes to dendritic degeneration (46). In acAD, we observed downregulation of multiple cytoskeletal proteins (Fig. 2) and changes in transport machinery consistent with axonal trafficking deficits. Such impairments are linked to synaptic failure and neurodegeneration during AD progression (47, 48). Because cytoskeletal architecture is tightly controlled by phosphorylation, the phosphosite changes detected in acAD, including those concordant with murine models, further support this mechanism (Figs. 5 and 6).

While our model introduces neuroinflammation and cellular aging, it retains the strengths of traditional coculture systems. Across both CL and acAD, we observed shared alterations in synaptic pathways and mitochondrial dynamics (Fig. 2, Fig. 3, Fig. 4). Synaptic dysfunction is central to progressive neurodegeneration, and defining effective repair strategies will be essential. Both models showed downregulation of core presynaptic vesicle-cycle components and changes in proteins involved in excitatory and inhibitory neurotransmitter handling, consistent with complex perturbations in neuronal signaling. We also detected broad effects on mitochondrial and metabolic proteins. Mitochondrial dysfunction is a feature of neurodegenerative disease and may constrain the energetic capacity required for sustained neuronal signaling (49). Finally, the cross-species concordance of phosphosite changes between acAD and murine models underscores the translational relevance of this model (Fig. 6).

### Phosphorylation Signatures

Phosphoproteomic profiling uncovered systemic shifts in RNA-binding and cytoskeletal proteins, with acAD phosphorylation patterns more closely aligning with murine AD models than protein abundance profiles alone (Fig. 5). These shifts were detected even without phosphopeptides enrichment in the original study design. Supporting our results, phosphorylation changes in acAD closely mirrored published phosphoproteomic data from two established murine AD models (33) (Fig. 6). Since phosphorylation regulates protein activity by modulating activation or silencing, these findings point to widespread functional changes beyond mere alterations in protein abundance. Phosphorylation plays a central role in AD pathogenesis, ranging from the hyperphosphorylation of tau by multiple kinases (10), to amyloid processing (50), to the regulation of amyloid precursor protein processing (50), and broader disruptions in kinase/phosphatase signaling pathways (51). This data suggests that phosphorylation captures the difference in functional/disease state better than protein levels alone for modeling AD. HCA clustering on protein levels alone (Supplemental Fig. S1) successfully grouped randomized samples according to their identifies (Nt, Pg, CL, and acAD) but not always in alignment with the disease stages. Particularly, the progression of AD symptoms is not linear but complex; for example, neurons are primarily hyperactive in the early stages of the disease and return to baseline or below baseline activity later on (40). It would be expected that proteins involved in these aspects of the disease are tightly regulated, not only at the level of expression, but also through dynamic post-translational modifications such as phosphorylation, which modulate protein function in a context-dependent manner. These modifications provide an added layer of regulation that may more accurately reflect functional disease states than protein abundance alone.

### Limitations

In co-culture, the inflammatory phenotype can arise from multiple cell types. Single-cell sequencing and spatial proteomics identified neuronal and glial subtypes that are especially vulnerable in AD (52, 53). Because our study used bulk TMT proteomics, cell-to-cell differences were averaged, and we cannot assign phosphosite or pathway changes to specific cell types, for example, sorting neurons, astrocytes, and microglia for proteome and phosphoproteome analysis, single-cell omics, and spatial proteomics. These approaches will test whether the neuroinflammatory program and cytoskeletal remodeling localize to defined subpopulations and will refine the mechanistic picture that emerges here.

### Translational Relevance

This integrated proteome–phosphoproteome resource positions acAD as a translationally relevant model that links cellular aging to AD phenotypes. It supports cross-species comparisons and provides a platform for testing therapeutic strategies that target cytoskeletal and inflammatory pathways. The molecular signatures identified here may aid future drug screening and testing, including approaches that target cytoskeletal derangement and inflammation to complement more traditional β-amyloid-based therapies. As cell-type-resolved methods are layered onto this system, the model should further improve in predictive value.

## Data Availability

The HRMS primary files and the reference human proteome were deposited to the ProteomeExchange Consortium via the PRIDE partner repository with the dataset identifier PXD062605.

## Supplemental Data

This article contains supplemental data.

## Conflict of interest

The authors declare no conflicts of interest.
