# Supplementary material for: Deep Profiling of the Aging Proteome Depicts Neuroinflammation, Synaptic Function, and Phosphorylation in an Accelerated Alzheimer's Disease Cell Model
Source: Mol Cell Proteomics. 2025 Dec 17;25(2):101490. doi: 10.1016/j.mcpro.2025.101490 (PMC12905760; doi:10.1016/j.mcpro.2025.101490)
Supplement: Supplemental Figures [file mmc1.pdf]

Supporting Information for

Deep Profiling of the Aging Proteome Depicts Neuroinflammation, Synaptic Function, and Phosphorylation in an Accelerated Alzheimer's Disease Cell Model

Emma Gentry[1], Md Tarikul Islam[2], Huijing Xue[1], Kan Cao[1*], and Peter Nemes[2*]

Departments of [1]Cell Biology & Molecular Genetics and
[2]Chemistry & Biochemistry, University of Maryland, College Park, MD, USA

***Correspondence to:** Peter Nemes, 0107 Chemistry Building, 8051 Regents Dr., College Park, MD 20742, USA, nemes@umd.edu, (Tel.) 301-405-0373; Kan Cao, 2114 Bioscience Research Building, College Park, MD 20742, USA, kcao@umd.edu, (Tel.) 301-405-3016.

TABLE OF CONTENTS

SI FIGURES 2
Figure S1 2
Figure S2 3
Figure S3 4
Figure S4 5
Figure S5 7
Figure S6 8
Figure S7 9
Figure S8 10

SI FIGURES

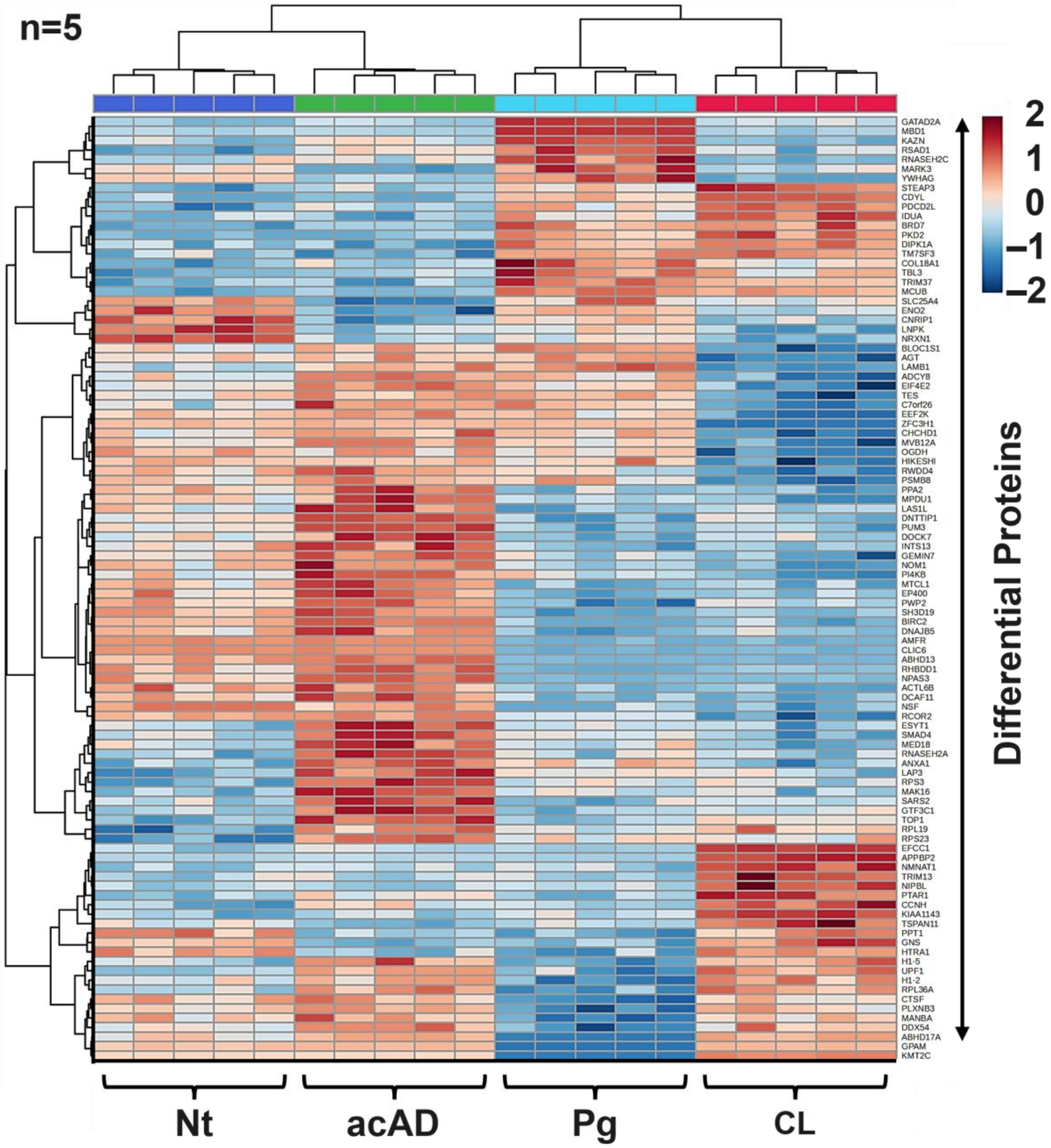

Figure S1. Close-up of **Figure 2A-B**. Hierarchical cluster-heatmap analysis on the statistically significant ($p < 0.05$) top 100 proteins quantified across biological replicates. Differential clustering reveals variations in protein translation dynamics among the classic (CL) and the accelerated (acAD) phenotype. The samples were grouped into Nt (control), Pg (Progerin), CL (classic), and acAD phenotypes. (Scale bar, Z scale)

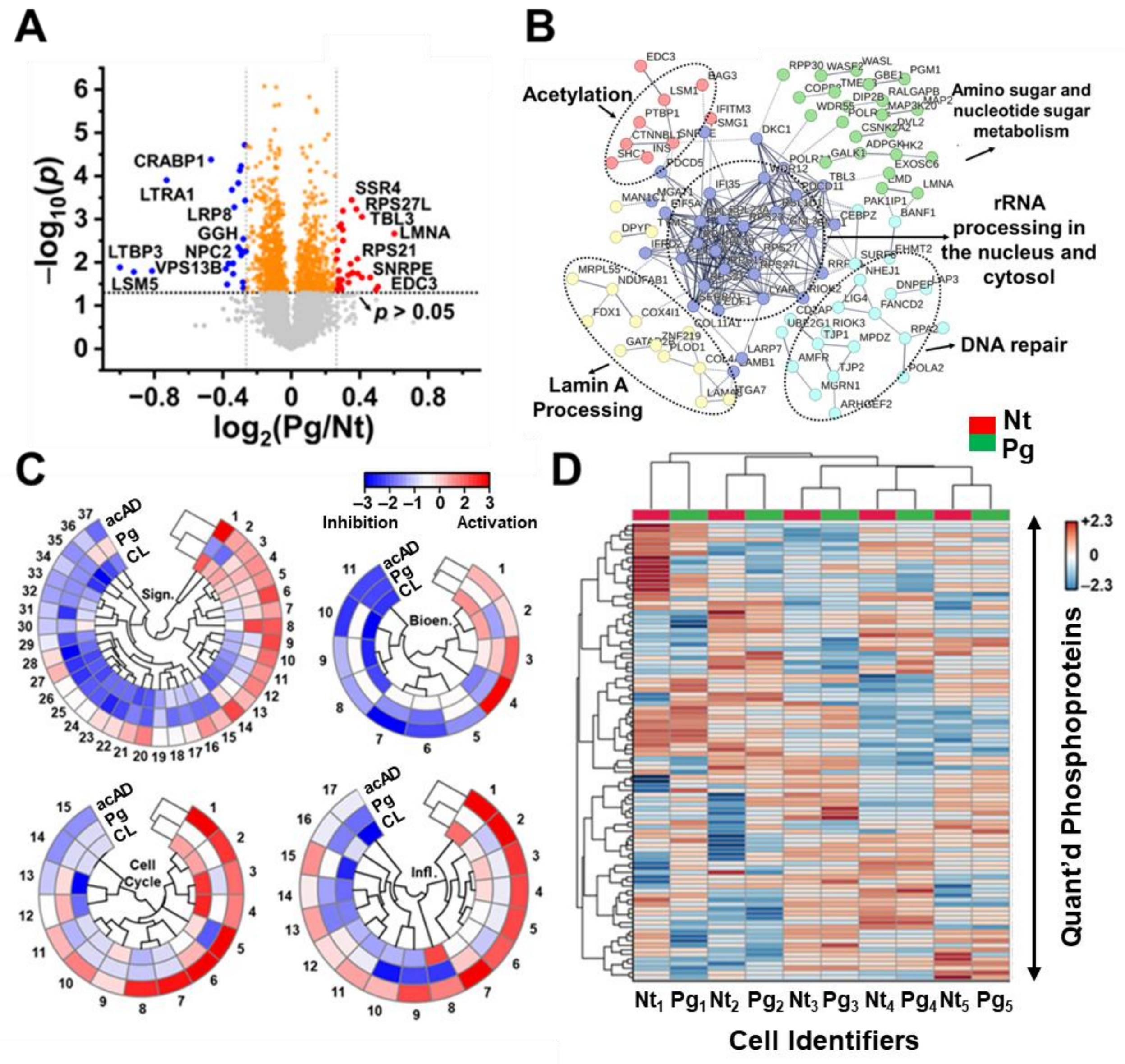

Figure S2. Progerin-only (Pg) control does not account for changes seen in acAD. (A) Differentially regulated proteins filtered by p < 0.05 and fold change > 1.2. **(B)** STRING predicted protein-protein interaction network shows focus points of various nuclear processes and metabolic changes. **(C)** Ingenuity pathway analysis reveals that changes in acAD (AppPg) differ from those seen in progerin-only control (Pg) and the classic model (CL). The key to the labels is available in **Table S5**. **(D)** Progerin phosphorylome is not distinguishable from the nontransduced control.

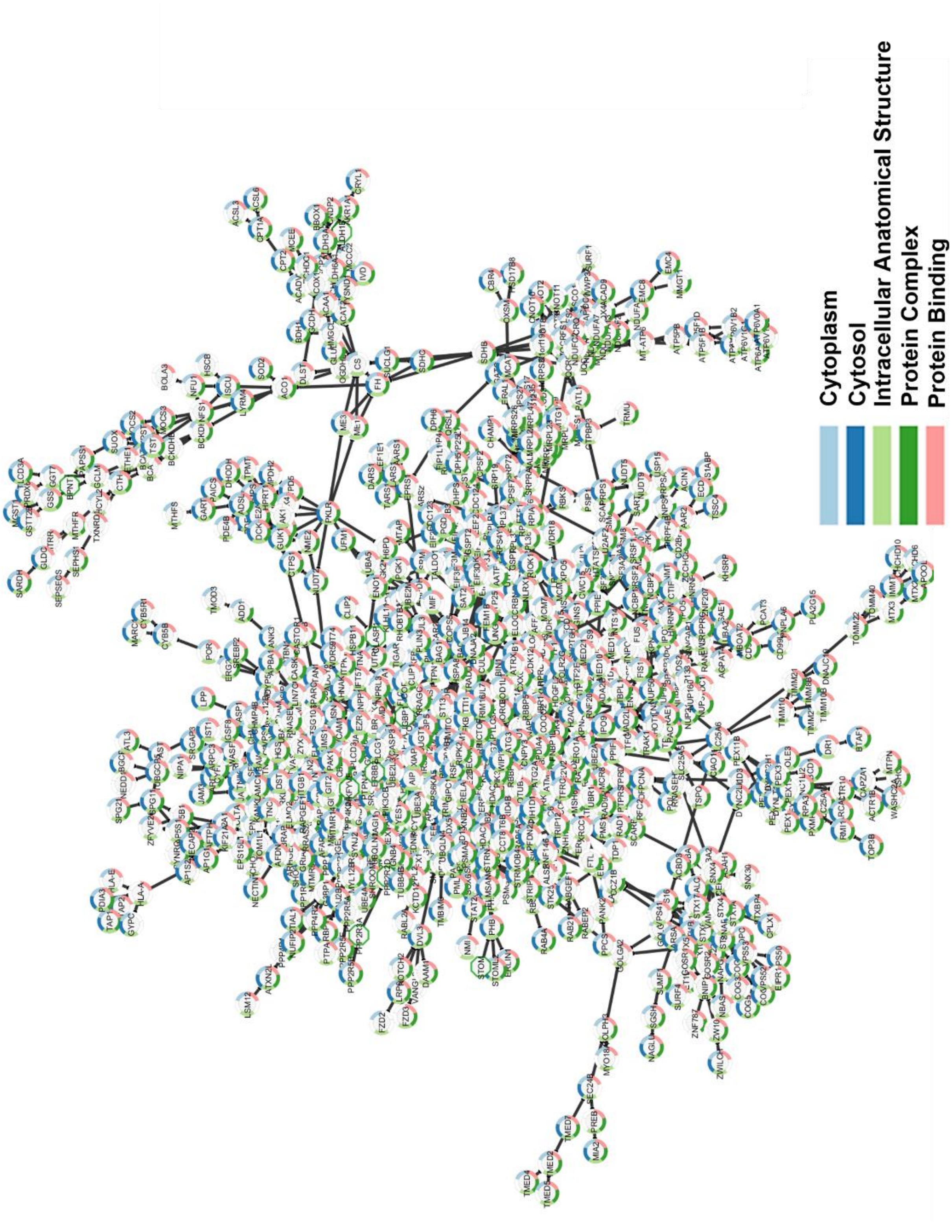

Figure S3. A close-up view of **Figure 3A** showing stable expression of cytoplasmic, and intracellular protein complexes in both CL and acAD (protein types are color-coded) with $p > 0.05$.

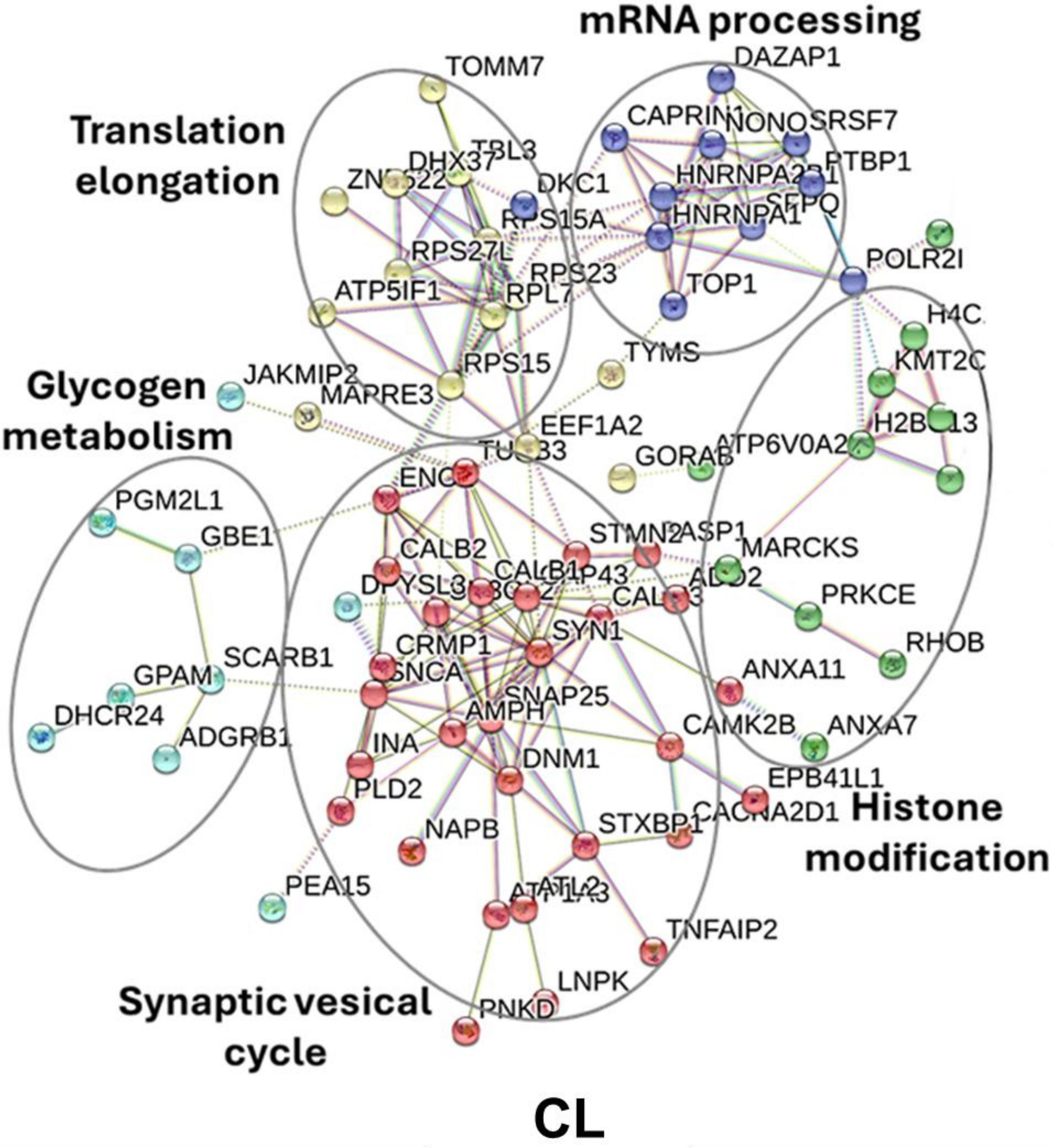

Figure S4: Classic Model. Close-up of **Figure 3B**, variable expression (fold changes cut off > 1.2 and $p < 0.05$, Student's t-test). These proteins supported diverse molecular pathways including synthetic vesical cycle, and mRNA processing commonly identified in CL and acAD. In contrast, the acAD model predicted bioenergetic pathways (oxidative phosphorylation, TCA metabolism), and pathways related to cytoskeletal rearrangement due to the aging factor. The Accelerated model is shown on the next page.

.

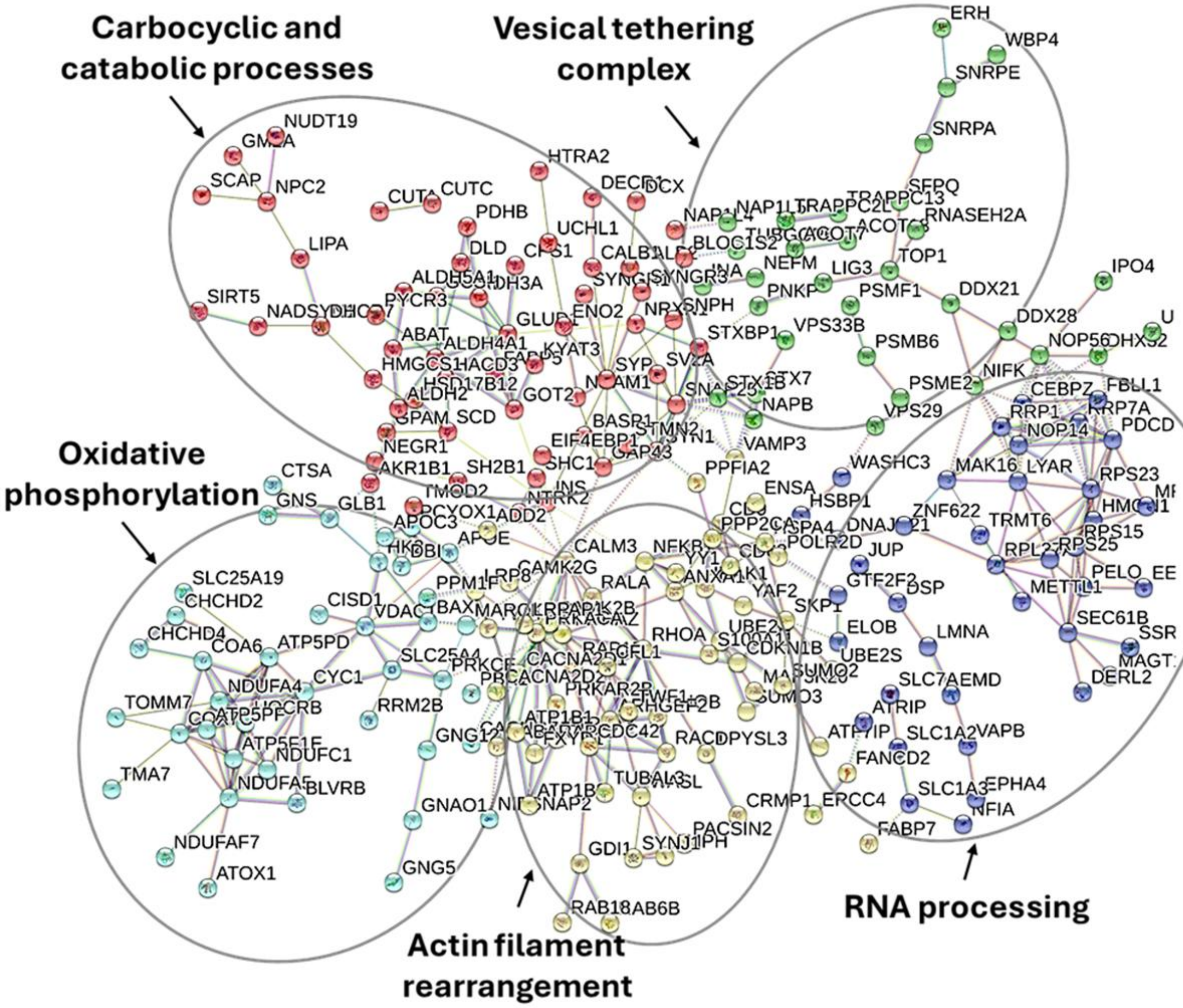

acAD

Figure S4: acAD. (The figure caption is on the previous page.)

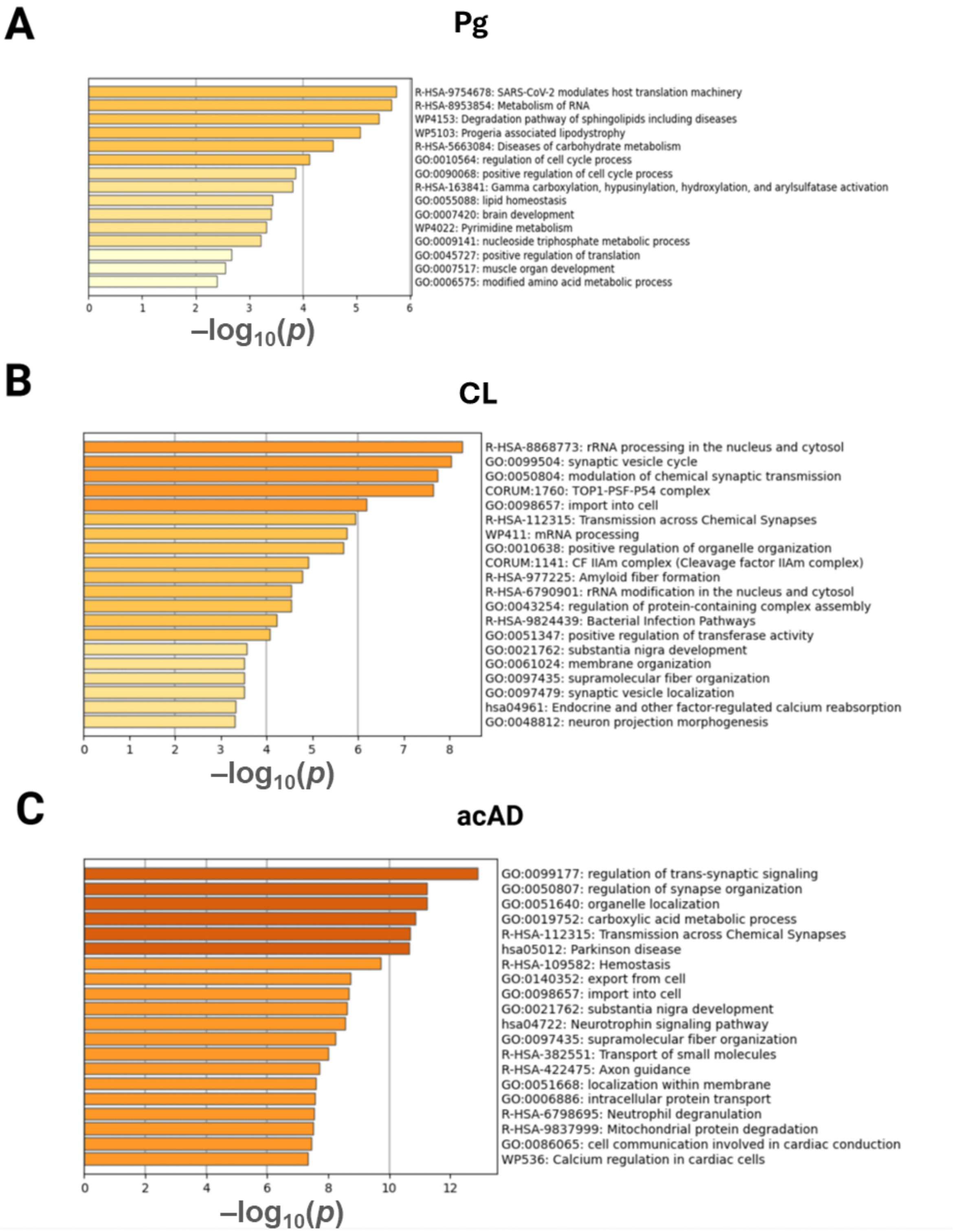

Figure S5. GO analysis on protein abundance level changes of $p < 0.05$ and fold change > 1.2 shows emphasis on neuronal function in both (**B**) CL and (**C**) acAD, and not in (**A**) the progerin-only condition. Graphs were generated using Metascape.

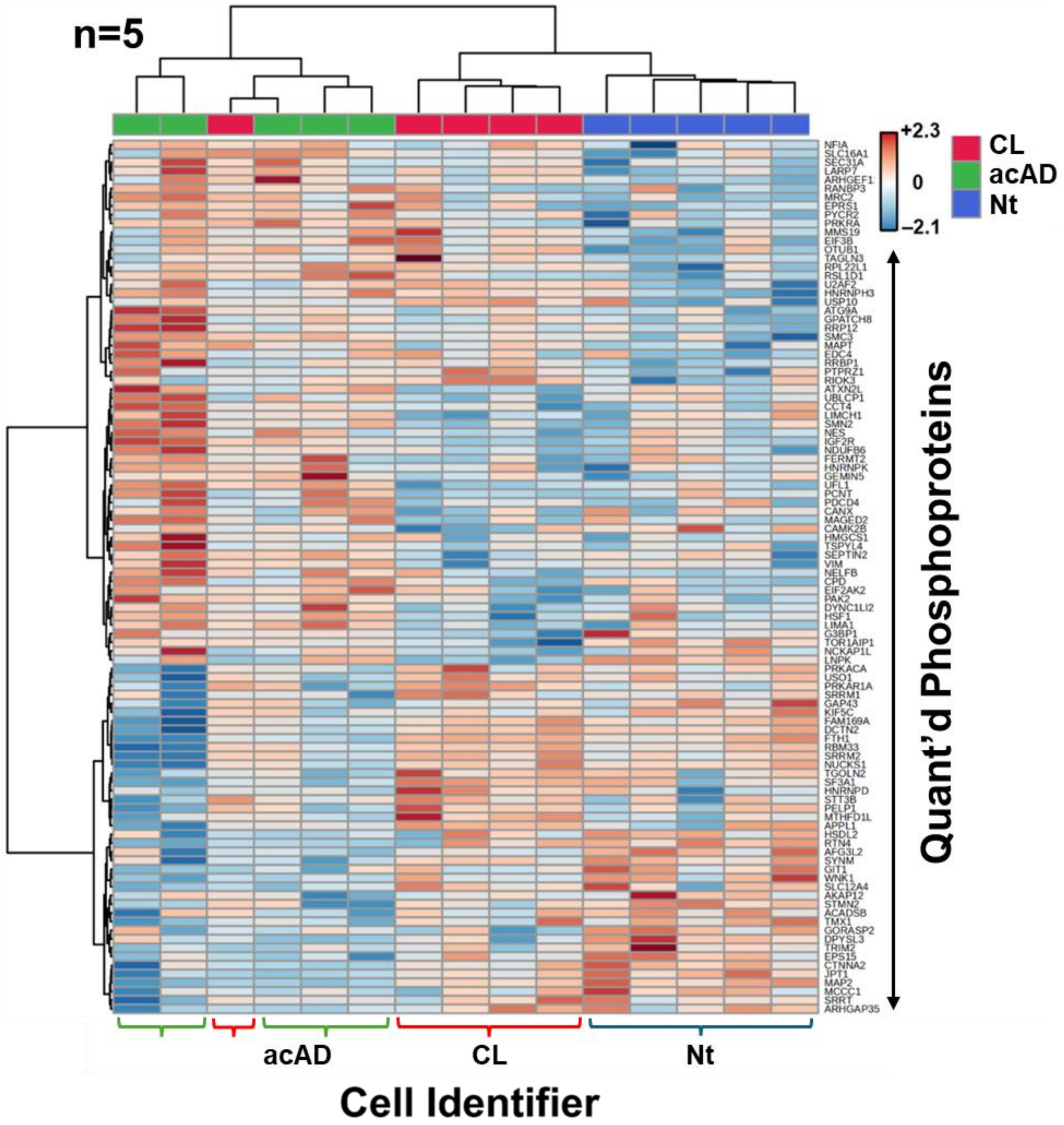

Figure S6. Close-up of the **Figure 5** hierarchical cluster-heatmap analysis on the top 100 most significantly differently quantified phosphopeptides (mapped to proteins), depicted systematic differences in phosphorylation dynamics (labels #1–4). The samples were grouped into the same Nt (control), CL, and acAD phenotypes. (Scale bar, Z scale)

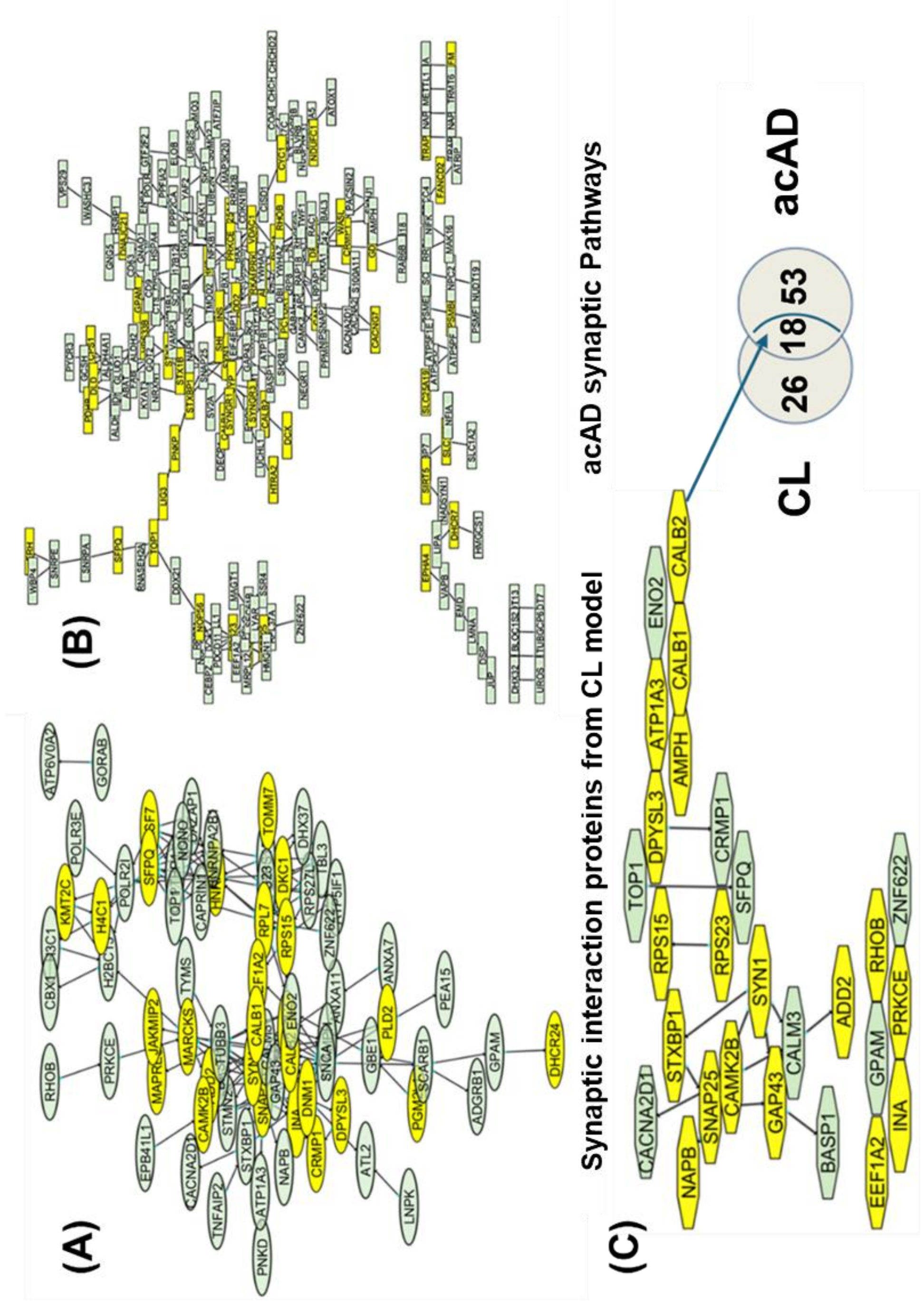

Figure S7. Both models show numerous synaptic proteins differentially phosphorylated**.** Network interactions shown for **(A)** CL and **(B)** acAD were generated to highlight phosphorylated synaptic proteins in yellow. **(C)** The overlapping proteins between both models are shown.

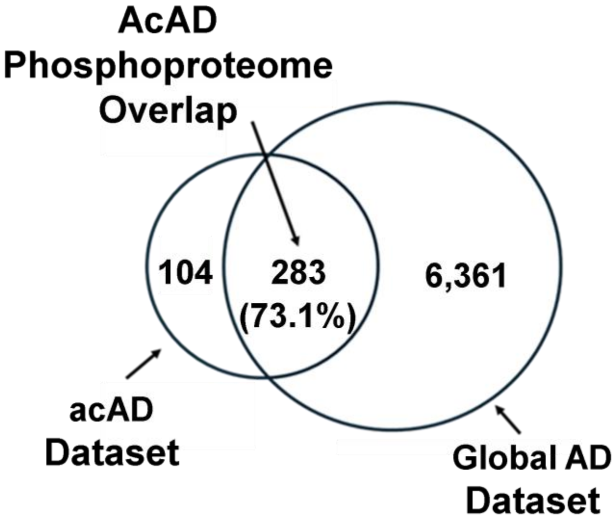

Figure S8. Comparison with Human AD Postmortem Phosphoproteomic Database. Published data on AD phosphoproteomics in postmortem samples were compared to the acAD phosphorylation data, where ~73% of the phospho-proteoforms in acAD model were found in the postmortem database.
